# Improvement of Insulin Resistance by *Lactobacillus johnsonii*-Derived Indole-3-Lactic Acid

**DOI:** 10.3390/microorganisms14061231

**Published:** 2026-05-30

**Authors:** Jie-Lin Zhan, Ya-Ting Wang, Yuan-Shan Yu, Yu-Juan Xu, Ji-Jun Wu, Jing Wen, Bo Zou, Hong Wang, Zhen-Lin Xu, Peng Wen, Teng-Gen Hu, Zhi-Bin Bu

**Affiliations:** 1 Guangdong Provincial Key Laboratory of Food Quality and Safety, College of Food Science, South China Agricultural University, Guangzhou 510642, China; paulinezzzz@163.com (J.-L.Z.); gzwhongd@163.com (H.W.); jallent@163.com (Z.-L.X.); pwen@scau.edu.cn (P.W.); 2 Sericultural & Agri-Food Research Institute, Guangdong Academy of Agricultural Sciences/Key Laboratory of Functional Foods, Ministry of Agriculture and Rural Affairs/Guangdong Key Laboratory of Agricultural Products Processing, Guangzhou 510610, China; wangyt4202@163.com (Y.-T.W.); yuyuanshan@gdaas.cn (Y.-S.Y.); wajijun@126.com (J.-J.W.); jingw988@163.com (J.W.); skzoubo@163.com (B.Z.); 3 School of Food Science and Engineering, South China University of Technology, Guangzhou 510641, China; 4 College of Food Science and Technology, Zhongkai University of Agriculture and Engineering, Guangzhou 510550, China; xyj6510@126.com

**Keywords:** *Lactobacillus johnsonii*, insulin resistance, gut microbiota, tryptophan metabolism, indole-3-lactic acid, mitochondrial complex IV, COX5B, oxidative stress

## Abstract

Insulin resistance (IR), a primary pathological driver of dysregulated glucose and lipid metabolism, is closely associated with elevated oxidative stress. Gut microbiota contributes to IR via bioactive metabolites. Among these, microbial tryptophan-derived indole compounds have emerged as key metabolic regulators, yet their specific targets remain unclear. In this study, 16S rRNA sequencing and metabolomics analyses indicated that tryptophan metabolism serves as the primary pathway through which *Lactobacillus johnsonii* Y1 alleviates IR. Genomic analysis of *L. johnsonii* Y1 and in vitro fermentation experiments subsequently identified indole-3-lactic acid (ILA) as the key functional metabolite. Further experimentation in a cellular IR model demonstrated that ILA restores insulin sensitivity and glucose uptake. Mechanistically, transcriptomic analysis revealed that ILA enhances mitochondrial complex IV (CIV) activity through the upregulation of COX5B, thereby reducing reactive oxygen species production, attenuating inflammation and restoring insulin signaling. Together, these findings highlight an *L. johnsonii*-derived ILA–CIV axis that alleviates oxidative stress and improves IR, offering a tryptophan–mitochondrial axis-targeted strategy for IR management.

## 1. Introduction

Insulin resistance (IR), characterized by diminished sensitivity to insulin, disrupts the regulation of glucose and lipid metabolism and serves as a core pathological driver of metabolic disorders such as type 2 diabetes and non-alcoholic fatty liver disease [1,2]. Growing evidence indicates the critical role of mitochondrial dysfunction and the resulting oxidative stress in the initiation and progression of IR [3,4]. Gut microbiota participates in host energy homeostasis and immune regulation through the production of various bioactive metabolites. Therefore, modulating the microbiota represents a promising but still emerging strategy to improve IR [5,6], given that clinical application is not yet established. Our preliminary findings revealed that *L. johnsonii* contributes to the maintenance of glucose and lipid homeostasis, yet the underlying mechanisms remain unclear.

As a precursor to a spectrum of microbial metabolites, the essential amino acid tryptophan gives rise to indole derivatives, which are increasingly recognized as crucial mediators, thereby linking gut microbial activity to host metabolic regulation [7,8,9]. Among these, indole-3-lactic acid (ILA) and indole-3-propionic acid (IPA) exert beneficial effects in terms of improving IR [8,10], attenuating oxidative stress, and reducing inflammation, thereby promoting systemic metabolic homeostasis [11,12]. Notably, recent studies have suggested a close association between *L. johnsonii* and the production of specific tryptophan-derived metabolites, particularly ILA, implicating the activation of this tryptophan metabolic pathway as a potential mechanism for its anti-IR properties [11,13,14,15].

Oxidative stress, a major driver of IR, is closely associated with impaired electron transport in mitochondrial dysfunction [16]. Mitochondrial respiratory chain complex IV (CIV), which is the terminal enzyme of the respiratory chain, preserves mitochondrial homeostasis by maintaining efficient electron transfer and limiting excessive reactive oxygen species (ROS) generation [17,18]. Therefore, dysfunction of CIV not only exacerbates oxidative stress and inflammation, but also actively promotes the progression of IR [19,20,21]. Based on the recognized antioxidant properties of ILA and IPA, we hypothesized that *L. johnsonii* may alleviate IR through the production of these tryptophan metabolites, likely by targeting and modulating CIV-associated pathways to reduce mitochondrial-related oxidative stress.

To test this hypothesis, the metabolic effects of *L. johnsonii* Y1 were systematically assessed using a high-fat diet-induced murine model of IR. Second, integrated 16S rRNA gene sequencing of feces and untargeted metabolomic profiling of serum were conducted to identify microbial and metabolic alterations associated with the intervention. Third, the genomic repertoire of *L. johnsonii* Y1 was analyzed to screen for genetic determinants involved in tryptophan metabolite synthesis. Finally, to dissect the host molecular response, transcriptomic analysis was performed on relevant metabolic tissues to investigate pathways related to mitochondrial function and oxidative stress. Our findings suggest a correlational *L. johnsonii*–ILA–CIV axis that improves IR and provides a hypothesis-generating foundation for future mechanistic validation.

## 2. Materials and Methods

### 2.1. Materials and Reagents

De Man-Rogsa-Sharpe (MRS) and Agar were purchased from HuanKai Microbial (027312, Guangzhou, China). Dulbecco’s modified Eagle’s medium (DMEM, 25 mM, 8122008, and 4.5 mM glucose, 6123034), fetal bovine serum (FBS, 2707023RP), trypsin—EDTA (0.25%, 8123673), penicillin–streptomycin (2585644), and phosphate-buffered saline (PBS, S123648) were obtained from Gibco Life Technologies (Carlsbad, CA, USA). Dimethyl sulfoxide (DMSO, HY-Y0320) and Cell Counting Kit 8 (CCK-8, HY-K0301) were sourced from MedChemExpress (South Brunswick, NJ, USA). 3-indole-propionic acid (IPA, BD13027) and 3-indole-lactic acid (ILA, BD13033) were purchased from Bide Pharmatech (Shanghai, China). Metformin hydrochloride (MET, B25331) was acquired from Yuanye Biotechnology (Shanghai, China). Glucosamine (GlcN, S1635), insulin (Y271393), intracellular ROS assay (S0036S), and glucose consumption assay kits (S0561S) were supplied by Beyotime Biotechnology (Shanghai, China). The glucose quantitative kits (A154-1-1), protein quantitative kits (A045-4-2), superoxide dismutase (SOD) assay kit (A001-3-2), and catalase (CAT) assay kit (A007-1-1) were obtained from Nanjing Jiancheng Bioengineering Institute (Nanjing, China). QuantiCyto^®^ Mouse/Rat Insulin ELISA kit was obtained from Neobioscience (EMRC001, Shenzhen, China). *TransScript*^®^ Uni All-in-One First-Strand cDNA Synthesis SuperMix for qPCR (One-Step gDNA Removal, AU341-02) and *PerfectStart*^®^ Green qPCR SuperMix (AQ602-02-V2) were purchased from TransGen Biotech (Beijing, China). E.Z.N.A. Stool DNA Kit (D4015-02) was provided by OMEGA Bio-Tek (Norcross, GA, USA). TruSeq™ DNA Sample Prep Kit was obtained from Illumina, Inc. (San Diego, CA, USA). RNA extraction kit was provided by Surbiopure Biotechnology (151602-48, Guangzhou, China).

### 2.2. Strain and Conditions of Culture

*L. johnsonii* Y1 (preserved at the Sericulture and Agri-Food Research Institution, Guangdong Academy of Agricultural Sciences, CICC No. 6084) was originally isolated from pig feces, which was publicly accessible through the China Center of Industrial Culture Collection (CICC). The strain was cultured anaerobically on MRS agar at 37 °C for 48h. Single colonies were then transferred to MRS broth and incubated anaerobically at 37 °C with shaking at 180 rpm for 24 h before use. For long-term preservation, strains were stored in 25% (*v*/*v*) glycerol at −80 °C after culturing, as described previously [22].

### 2.3. Animal Experiments

All animal procedures were approved by the institutional Animal Care and Use Committee to the Sericultural and Agri-Food Research Institute, Guangdong Academy of Agricultural Sciences (Ethics No. SYXK [Yue] 2020-0149). Male C57BL/6J mice (4 weeks old) were group-housed under specific pathogen-free (SPF) conditions in individually ventilated cages (IVCs), maintained at 24 ± 2 °C with a 12 h light/dark cycle. Water, food and the bedding were sterilized before being supplied. The health status of the animals was monitored in accordance with the Chinese national standard GB 14922-2022 [23] and the ARRIVE guidelines for laboratory animal microbiological and parasitological quality control. After one week of accommodation, mice were randomly divided into 4 groups (6 per cage, one cage per group). Normal control (NC) were fed a basal diet (BD) and intragastrically gavaged with saline for 6 weeks. The model control group (MD) was fed a high-fat diet (HFD) and gavaged daily with saline for 6 weeks, and the *L. johnsonii* Y1-treated group (*L. johnsonii*) was fed HFD and gavaged daily with 2 × 10^9^ CFU/kg·bw *L. johnsonii* Y1 for 6 weeks, while the positive control group (PC) was fed HFD and gavaged daily with 50 mg/kg·bw metformin for 6 weeks. Fasting blood glucose was monitored weekly. Fresh feces were collected directly from each mouse using a sterile tube in the morning on three consecutive days prior to euthanasia, snap-frozen in liquid nitrogen within 30 min, and stored at −80 °C. An amount of 100mg was taken from each sample, added into a lysing matrix E tube together with 978 µL sodium phosphate buffer and 122 µL MT buffer, and homogenized in an MP homogenizer at 6 m/s for 40 s before metabolomic analysis.

At the end of the intervention, mice were fasted for 12 h, followed by anesthesia with isoflurane (4%, *v*/*v*) and euthanasia by carbon dioxide asphyxiation at a flow rate of 30–70% chamber volume per minute. The CO_2_ flow was maintained for 2 min after respiratory arrest, followed by cervical dislocation to ensure death. Serum and intestinal contents were collected for further analysis.

### 2.4. Oral Glucose Tolerance Test (OGTT) and Insulin Tolerance Test (ITT)

For OGTT, mice were fasted for 12 h (water allowed) and then administered glucose (2 g/kg body weight) by gavage. For ITT, mice were fasted for 6 h and injected intraperitoneally with insulin (0.75 IU/kg body weight). Blood glucose was measured at 0, 30, 60, 90 and 120 min using a glucometer (Omron, Kyoto, Japan). The area under the curve (AUC) was calculated to quantify both tests.

### 2.5. Assessment of the Insulin-Resistant Index

After an overnight fast, blood was collected for measurement of fasting glucose (as described previously in this research) and fasting insulin (using a commercial ELISA kit). The homeostatic model assessment–insulin resistant (HOMA-IR) index was calculated using the equation: HOMA-IR = Fasting insulin (mIU/L) × Fasting glucose (mM)/22.5.

### 2.6. 16S rRNA and Serum Metabolomic Sequencing and Data Analysis

Feces and serum from mice were collected for 16S rRNA and metabolomic sequencing. These analyses were performed by Majorbio Bio-pharm Technology Co. (Shanghai, China).

Specifically, genomic DNA for 16s rRNA sequencing was directly extracted with a stool DNA kit and assessed by 1% agarose gel electrophoresis. Using the 338-F and 806-R primer pair, the V3–V4 hypervariable region of the 16S rRNA gene was PCR amplified. PCR products were quantified using the QuantiFluor™-ST system (Promega Corporation, Madison, WI, USA), and pooled equimolarly, and libraries were constructed with the TruSeq™ DNA Sample Prep Kit. Sequencing was then performed on the Illumina platform using standard cluster generation and sequencing-by-synthesis protocols. For bioinformatics, the optimized reads were clustered into operational taxonomic units (OTUs) at a 97% sequence similarity threshold using UPARSE v7.1 (no ASV denoising was applied). Taxonomy was assigned with the RDP Classifier v2.2 against the Silva v138 database at a confidence threshold of 0.7.

For untargeted metabolomics, 100 mg feces were homogenized for 6 min (−10 °C, 50 Hz) in 800 μL methanol/water (4:1, *v*/*v*) containing four internal standards (e.g., L-2-chlorophenylalanine, 0.02 mg/mL) using a 6 mm steel bead. After ultrasonication for 30 min (5 °C, 40 kHz), samples were incubated at −20 °C for 30 min, and the supernatants were transferred to autosampler vials for analysis via centrifugation (4 °C, 13,000× *g*, 15 min). LC–MS analysis was performed on a Thermo Scientific UHPLC–Orbitrap Exploris 240 system, and a 3 µL aliquot was injected onto an HSS T3 column (100 × 2.1 mm, 1.8 μm). Mobile phase A was water/acetonitrile (95/5, *v*/*v*) with 0.1% formic acid, and mobile phase B was acetonitrile/isopropanol/water (47.5/47.5/5, *v*/*v*/*v*) with 0.1% formic acid. The flow rate was 0.40 mL/min and the column temperature was 40 °C. Mass spectrometry was conducted in both positive and negative ion modes over m/z 70–1050. Spray voltages were +3500 V and –3000 V, with sheath gas 50 arb, auxiliary gas 13 arb, ion source temperature 450 °C, and a stepped collision energy of 20/40/60 eV. The data were further processed by bioinformatics pipelines on the Majorbio Cloud Platform.

### 2.7. Whole Genome Analysis of L. johnsonii Y1

Genomic DNA was extracted from *L. johnsonii* Y1 cultures using a commercial kit. DNA purity and concentration were assessed with a Nanodrop spectrophotometer (Thermo Fisher Scientific, Waltham, MA, USA) and Qubit fluorometer (Invitrogen, Carlsbad, CA, USA). In addition, DNA integrity was evaluated by 0.35% agarose gel electrophoresis. To obtain long fragments suitable for sequencing, high-molecular-weight DNA fragments were then selected using a BluePippin system (Sage Science, Beverly, MA, USA). Libraries were prepared with the SQK–LSK ligation sequencing kit and sequenced on an Oxford Nanopore platform. The resulting long reads were assembled and annotated for downstream analysis.

### 2.8. Quantitation of ILA and IPA

Culture supernatants were extracted three times with two volumes of ethyl acetate. The combined organic phases were evaporated under reduced pressure at 60 °C and the residue was redissolved in methanol (one-tenth of the original volume). After filtration through a 0.22 μm organic membrane, samples were analyzed by high-performance liquid chromatography (HPLC, Shimadzu, Kyoto, Japan) on a C18 reversed-phase column (4.6 × 200 mm, 5 μm). The mobile phase consisted of 0.05% trifluoroacetic acid in water (A) and acetonitrile (B) with a gradient from 10% A/90% B to 13.8% A/86.2% B over 5 min. Flow rate was 1 mL/min and injection volume was 10 μL.

### 2.9. Cell Cultures and Treatments

#### 2.9.1. Cell Cytotoxicity Tests

First, HepG2 cells (human hepatoma cell line, BNCC, Zhengzhou, China) were maintained in DMEM with 25 mM glucose, supplemented with 10% FBS and 1% penicillin–streptomycin, at 37 °C in a 5% CO_2_ humidified atmosphere. In experimental groups, HepG2 cells were seeded in 96-well plates with a density of 1 × 10^4^ cells/well. After 24 h, the medium was replaced by serum-free DMEM (4.5 mM glucose) containing various concentrations of GlcN (0, 4.5, 9, 18, and 36 mM), IPA, ILA, or MET (0, 100, 200, 400, and 800 μM). After 24 h of treatment, cells were washed with PBS twice and incubated with CCK-8 solution (10% in serum-free DMEM with 4.5 mM glucose) for 2 h at 37 °C. Absorbance was measured at 450 nm by a microplate reader (Infinite 200 PRO, Tecan, Mannedorf, Switzerland).

#### 2.9.2. Establishment and Verification of Insulin-Resistant HepG2 Cell (IR-HepG2) Models

HepG2 cells were seeded in 12-well plates (5 × 10^5^ cells/well). After 24 h, IR was induced by incubating cells with 18 mM GlcN in serum-free DMEM (4.5 mM glucose) for 18 h. Cells were then stimulated with 100 nM insulin for 20 min, and glucose consumption was measured using a commercial kit to confirm the IR phenotype.

#### 2.9.3. Glucose Uptake Assay

IR-HepG2 cells were incubated in serum-free DMEM supplemented with 4.5 mM glucose and treated as follows for 24 h and with three biological replicants set in each group. The model control (MD) group was treated with PBS solution, while the positive model was incubated with 400 μM MET. The IPA group was treated with 400 μM IPA, and the ILA group was treated with 400 μM ILA. Normal HepG2 cells treated with PBS served as the normal control (NC). Following incubation, cells were treated with or without 100 nM insulin for 20 min, and then exposed to 40 μM 2-[N-(7-Nitrobenz-2-oxa-1,3-diazol-4-yl) amino]-2-deoxy-d-glucose (2-NBDG) for 45 min at 37 °C. The cells were rinsed with PBS, and fluorescent images were captured using a Nikon Eclipse Ts2-FL microscope (Nikon, Melville, NY, USA) at 10× magnification.

#### 2.9.4. Intracellular ROS Levels

Following the treatment described earlier in Section 2.9.3, cells were incubated with 10 μM DCFH-DA for 20 min. After incubation, the cells were washed with PBS, and the fluorescent images were captured using a Leica TCS SP8 STED 3X microscope (LEICA, Wetzlar, Germany) at 10× magnification. Fluorescence intensity was then quantified using ImageJ software.

#### 2.9.5. Intracellular SOD and CAT Activities Assay

For the enzymatic assays, cells were scraped into 300 μL PBS, and the intracellular anti-oxidase activities (SOD and CAT) were determined using kits following the manufacturer’s instructions. Additionally, the protein concentration in the cells was assessed using the BCA assay kit, and enzyme activities were normalized to total protein.

### 2.10. Extraction of RNA and RT-qPCR Analysis

Total RNA was extracted using a commercial kit, and RNA concentration was measured with a Nanodrop spectrophotometer (Thermo Fisher, Waltham, MA, USA). Complementary DNA (cDNA) was synthesized from 1 μg RNA using TransScript^®^ SuperMix. qPCR was performed with PerfectStart^®^ Green SuperMix on a QuantStudio 3 (Thermo Fisher, USA). Gene expression was normalized to *β-ACTIN*. Primers are listed in Supplementary Table S1.

### 2.11. RNA Sequencing and Data Analysis

HepG2 cells were cultured and treated as described in Section 2.9. Total RNA was extracted from cells treated with different conditions described in Section 2.10 (each sample was collected from one well of the 12-well plate containing 5 × 10^5^ cells). The control group was the IR-HepG2 model group. RNA integrity was assessed using an Agilent 2100 Bioanalyzer (Agilent Technologies, Inc., Santa Clara, CA, USA), and all samples had an RNA integrity number (RIN) ≥ 8.0. Libraries were prepared using the MGIEasy RNA Library Prep Kit (MGI, Shenzhen, China) with polyA selection. Paired-end sequencing (2 × 100 bp) was performed on an MGI DNBSEQ-T7 platform, generating approximately 30 million raw reads per sample, obtained by Tsingke Biotech Co. (Beijing, China). Differentially expressed genes were identified and further processed by bioinformatics pipelines on the Tsingke Cloud Platform. Specifically, differential expression analysis was performed using Hisat2 v2.2.1 (alignment to GRCh38/hg38), FeatureCounts v2.0.3 (quantification), and DESeq2 v1.38.3 (|log_2_FC| ≥ 1, FDR < 0.05).

### 2.12. Statistical Analysis

Data were presented as the mean ± standard deviation (SD). For metabolomic data, the Wilcoxon rank-sum test was used on the Majorbio Cloud Platform, and *p*-values were adjusted for multiple comparisons using the Benjamini–Hochberg false discovery rate (FDR); metabolites with FDR < 0.05 were considered significantly altered. For RNA-seq, differential expression was analyzed using DESeq2 v1.38.3 with thresholds of |log_2_FC| ≥ 1 and FDR < 0.05. All other comparisons were considered statistically significant at *p* < 0.05. Other statistical analyses were performed using SPSS 21.0 (IBM Corp., Armonk, NY, USA). The assumptions of normality and homogeneity of variance were formally tested using the Shapiro–Wilk test and Levene’s test, respectively. As all data met these assumptions (*p* > 0.05), one-way ANOVA followed by Tukey’s HSD post-hoc test was used. A *p* value < 0.05 was considered statistically significant.

## 3. Results

### 3.1. Effect of L. johnsonii Y1 on Alleviation of IR and Remodeling Gut Microbiota

**Figure 1:**
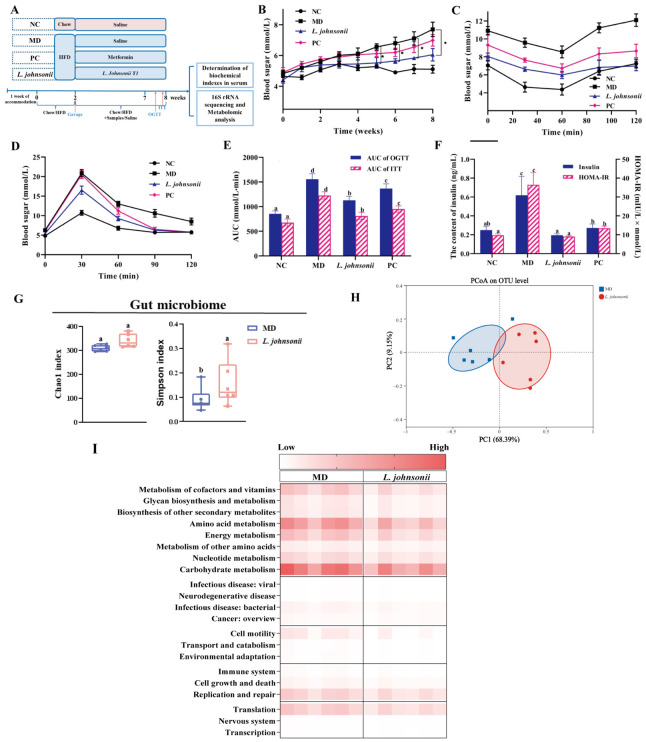
Effect of *L. johnsonii* on improving IR and its regulatory function in gut microbiome. (**A**) Schematic diagram depicting *L. johnsonii* intervention timeline in HFD-induced IR, (**B**) fasting blood glucose levels during the 8-week intervention, (**C**) oral glucose tolerance test (OGTT), (**D**) insulin tolerance test (ITT) curves, (**E**) area under the curve (AUC) of OGTT and ITT, (**F**) serum insulin concentration and HOMA-IR index at week 8, (**G**) Chao1 and Simpson index, (**H**) PCoA plot and (**I**) heatmap of KEGG level 2 functional pathways predicted by PICRUSt2 after *L. johnsonii* Y1 intervention (*p* < 0.05). The corresponding level 1 classifications are shown on the left. Data in (**B**–**F**) are presented as means ± SD from six mice per group. Different letters in the lower case and * represent significant difference among groups (*p* < 0.05).

To evaluate the effect of *L. johnsonii* Y1 on IR, key metabolic parameters were evaluated (Figure 1A). Compared with the MD group, administration of *L. johnsonii* Y1 significantly lowered fasting blood glucose from week 5 to week 8, as well as the AUC of both OGTT and ITT, serum insulin levels, and the HOMA-IR index at week 8 (all *p* < 0.05), indicating improved glucose tolerance and insulin sensitivity (Figure 1B–F). Analysis of fecal microbiota via 16S rRNA sequencing revealed that *L. johnsonii* Y1 treatment increased microbial α-diversity, as reflected by the Chao1 and Simpson indices, and altered the overall community structure (β-diversity), as visualized by principal coordinate analysis (PCoA) (Figure 1G,H). Functional prediction by PICRUSt2 further identified 21 KEGG pathways that were significantly enriched (FDR-corrected *p* < 0.05), among which carbohydrate metabolism and amino acid metabolism were the most prominent categories (Figure 1I).

### 3.2. Tryptophan Metabolism as the Key Pathway Upon L. johnsonii Y1 Intervention

**Figure 2:**
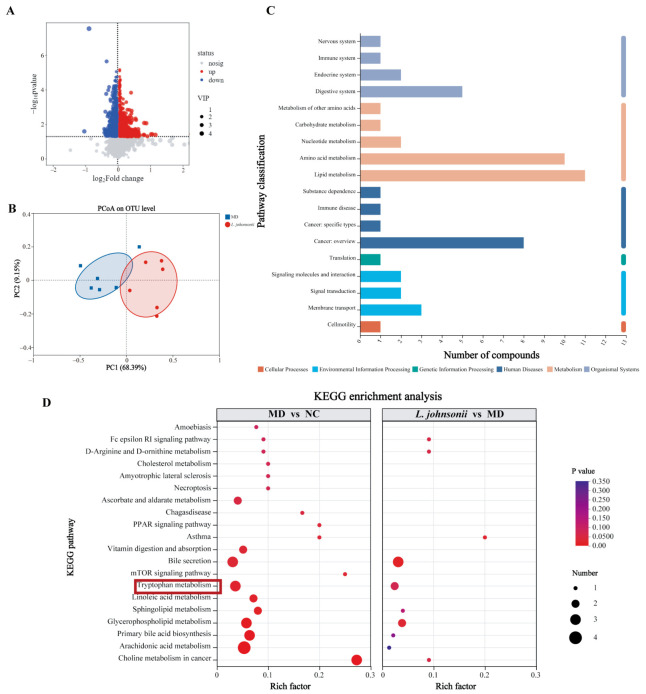
Metabolomic analysis of *L. johnsonii* intervention. (**A**) Volcano plot, (**B**) PLS-DA plots, (**C**) Summary of KEGG classification, and (**D**) bubble plot of KEGG enrichment analysis showing the top 20 significantly enriched pathways for the *L. johnsonii* vs. the MD and the MD vs. the NC groups, respectively. The color of the bubble gradient from red to blue represents decreasing significance (increasing *p*-value).

Untargeted serum metabolomic profiling revealed a total of 76 significantly altered metabolites, with 23 upregulated and 53 downregulated (Figure 2A), accompanied by a clear separation between the MD and the *L. johnsonii* Y1-treated group in partial least squares-discriminant analysis (PLS-DA) score plot (Figure 2B). Annotation of these differential metabolites to KEGG pathways indicated that the predominant enrichment occurred in amino acid metabolism and lipid metabolism (Figure 2C), which was consistent with the functional predictions from 16S rRNA sequencing via PICRUSt2.

To pinpoint the key pathways altered by HFD and subsequently modulated by the treatment, the top 20 pathways enriched in the *L. johnsonii* group (vs. the MD group) were analyzed in parallel with the enrichment pattern of these same pathways in the MD group (vs. the NC group) (Figure 2D). It was identified that the tryptophan metabolism, choline metabolism in cancer, glycerophospholipid metabolism, asthma, and bile secretion (*p* < 0.05) were the major pathways responsible for differentiating the NC and MD groups, serving as key mediators of the HFD-induced metabolic disturbance. Notably, while multiple pathways exhibited significant changes, tryptophan metabolism was the only significantly enriched pathway classified under amino acid metabolism, suggesting that it may represent the primary metabolic pathway affected by the intervention. Hence, based on the above metabolomic profiling, it can be concluded that tryptophan metabolism was the most prominently altered pathway upon *L. johnsonii* Y1 intervention.

### 3.3. Genomic and Functional Basis of ILA Biosynthesis by L. johnsonii Y1

**Figure 3:**
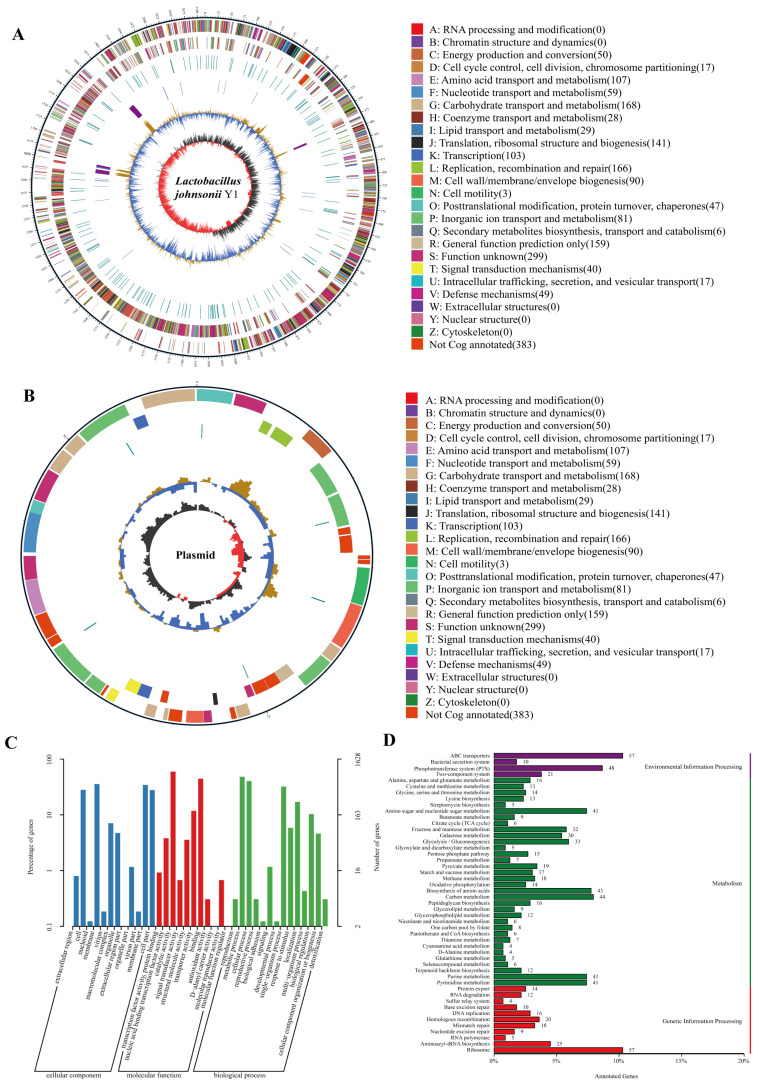
Genome analysis of *L. johnsonii* Y1. (**A**) Circular genome map of chromosome and (**B**) plasmid. (**C**) GO annotation and (**D**) KEGG annotation of *L. johnsonii* Y1.

To establish a comprehensive understanding of the genetic basis underlying ILA production and IR alleviation, we first performed a global characterization of the *Lactobacillus johnsonii* Y1 genome. The complete genome of *L. johnsonii* Y1 comprises a single circular chromosome (2114452bp) and a linear plasmid (57452bp), encompassing a total of 4290 predicted coding genes. Among these, 4189 genes were located on the chromosome and 101 on the plasmid (Figure 3A,B, and Supplementary Table S2). Based on Gene Ontology (GO) classification, these genes were categorized into 12 groups associated with cellular components, 11 to molecular functions, and 14 to biological processes (Figure 3C). Additionally, KEGG pathway annotation further classified these genes into three primary functional classes, with metabolism representing the largest category, encompassing 1146 genes (Figure 3D).

**Figure 4:**
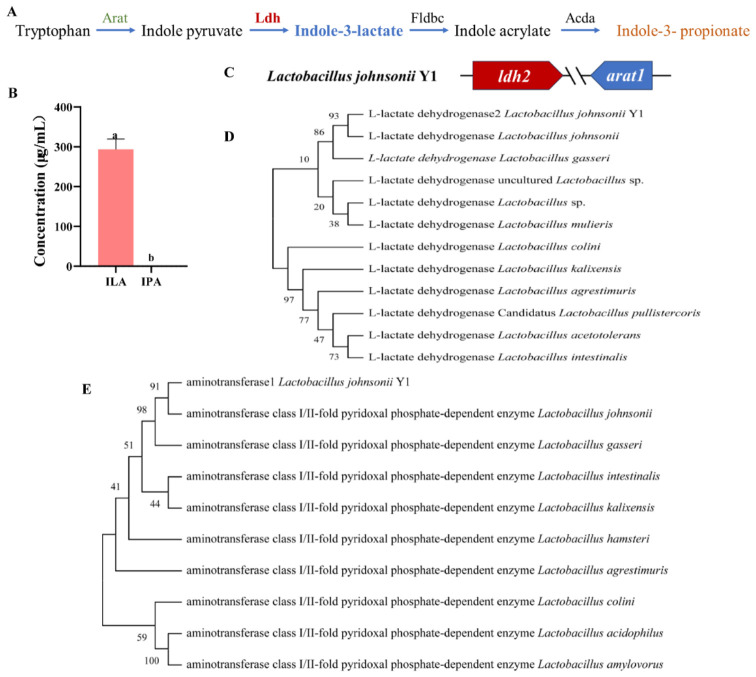
Genome analysis of *L. johnsonii* Y1 in Trp metabolism. (**A**) Schematic diagram depicting catalyzation of tryptophan in bacteria. (**B**) Ability of producing IPA and ILA of *L. johnsonii* Y1. (**C**) The possible gene organization of ILA production. (**D**) Neighbor-joining phylogenetic tree of Ldh2 and (**E**) Arat1. All data are presented as means ± SD from at least three independent experiments. Different letters in the lower case represent significant difference among groups (*p* < 0.05).

Given the prominence of tryptophan metabolism in metabolomic results, the genes involved in tryptophan catabolism were examined. In this pathway, tryptophan is initially converted to indole pyruvate (IPYA) by aromatic amino acid aminotransferase (Arat), and is subsequently reduced to ILA via lactate dehydrogenase (Ldh). In contrast, the biosynthesis of IPA requires additional enzymatic steps involving flavodoxin B/C (Fldbc) and acyl-CoA dehydrogenase (Acda), which were not identified in the genome (Figure 4A) [10]. Genomic mining of *L. johnsonii* Y1 identified two putative *arat* genes and three *ldh* gene candidates (Supplementary Table S3).

To verify the functional predictions from genomic analysis, ILA and IPA levels in bacterial culture supernatants were quantified using HPLC. Consistent with the genomic content, HPLC quantification revealed measurable production of ILA but no detection of IPA (Figure 4B), confirming that *L. johnsonii* Y1 primarily generates ILA rather than IPA.

Phylogenetic reconstruction is a widely utilized method to confirm gene homology and infer potential functional conservation among related species [24]. Consequently, neighbor-joining phylogenetic trees of *ldh2* and *arat1* were constructed to validate this prediction and genetically support our hypothesis. As shown in Figure 4D,E, *arat1* and *ldh2* genes are conserved across *Lactobacillus* species. The deduced Arat1 protein shares 91% amino acid identity with characterized Arat (EC 2.6.1.1), suggesting that Arat1 likely possesses high affinity for tryptophan. Similarly, Ldh2 exhibited 93% identity to functional Ldh (EC 1.1.1.27). Together, these results provide genetic evidence supporting the capacity of *L. johnsonii* Y1 to convert tryptophan into ILA.

### 3.4. Effects of ILA on Insulin Sensitivity and Oxidative Stress in HepG2 Cells

To investigate the underlying mechanisms of ILA on IR cells, the cytotoxicity tests of MET, IPA, and ILA were first assessed using the CCK-8 assay to determine the suitable working concentrations (Figure S1). Cell viability remained above 90% for all three compounds at concentrations ranging from 100 to 800 µM. However, increasing the concentration of IPA or MET from 400 to 800 µM led to a reduction in cell viability, with IPA showing a decrease of approximately 40% (*p* < 0.05), whereas ILA showed no significant cytotoxicity across the entire concentration range (*p* > 0.05). Based on these results, and considering the roles of IPA (indole compound control) and MET (positive control), a concentration of 400 µM was selected for subsequent experiments to ensure both biological activity and minimal cellular toxicity.

**Figure 5:**
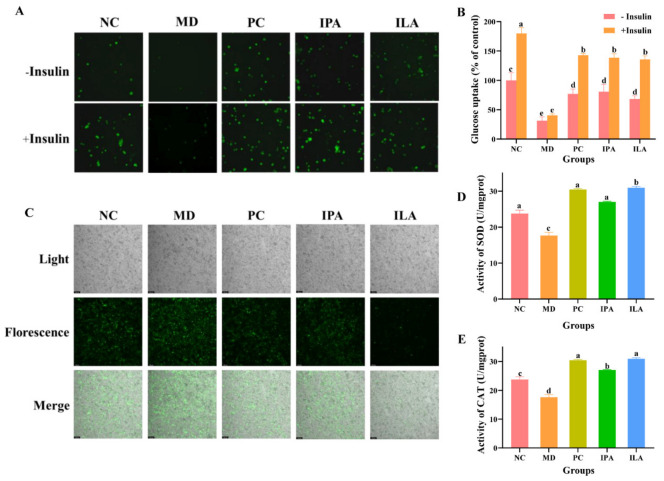
Effect of ILA on the glucose uptake oxidative stress in HepG2 cells. (**A**) The fluorescent image of 2-NBDG in HepG2 cells was captured at 10× magnification. (**B**) Glucose uptake in HepG2 cells with or without insulin stimulation. (**C**) Representative images of ROS level-derived DCFH-DA green fluorescence of HepG2 cells. (**D**) Activity of SOD in HepG2 cells. (**E**) Activity of CAT in HepG2 cells. All data are presented as means ± SD from at least three independent experiments. The scale bar is 100 μm in the fluorescence figures. Different letters in the lower case represent significant difference among groups (*p* < 0.05).

Subsequently, the effect of ILA on insulin-stimulated glucose uptake in HepG2 cells was evaluated using 2-NBDG and glucose consumption assays. As shown in Figure 5A,B, GlcN treatment markedly inhibited insulin-stimulated glucose uptake, confirming the successful induction of IR. Treatment with MET, IPA, and ILA significantly reversed this inhibition with an enhancement of glucose utilization compared with the MD group, with no significant differences observed among the three treatments.

Given the close association between oxidative stress and IR, the levels of intracellular ROS and the activities of key oxidative-related enzymes were measured. As shown in Figure 5B, ILA visibly reduced ROS production. Correspondingly, the activities of oxidative-related enzymes (including SOD and CAT) were elevated following interventions with MET, IPA, and ILA (Figure 5C–E, *p* < 0.05), in which, when compared with IPA, ILA exhibited a greater effect in alleviating oxidative stress (*p* < 0.05)**.** These results suggest that the amelioration of IR by ILA may be linked to its capacity to mitigate oxidative stress.

### 3.5. ILA-Mediated Activation of the AHR-COX5B Axis to Mitigate Oxidative Stress

**Figure 6:**
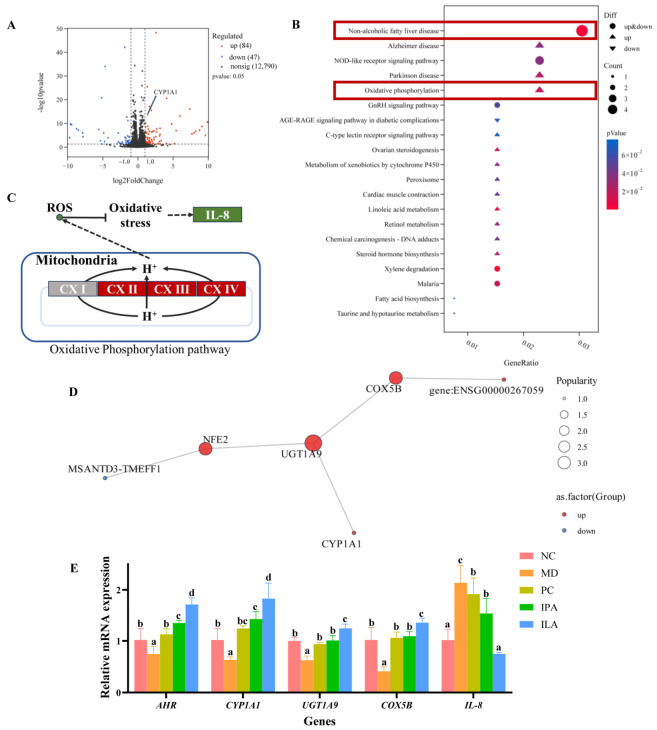
Transcriptome profiling of HepG2 cells. (**A**) Volcano plots of DEGs in HepG2 cells (blue, downregulated; red, upregulated; change > 2-fold, *p* < 0.05). (**B**) Bubble plots showing DEGs in the top 20 KEGG pathways. (**C**) Part of non-alcoholic fatty liver disease (NAFLD, term 04932) in the KEGG database (green, downregulated; red, upregulated). (**D**) Protein–protein interaction (PPI) network of key DEGs. (**E**) Relative mRNA expression of key genes identified in RNA sequencing including *AHR*, *CYP1A1*, *UGT1A9*, *COX5B*, and *IL-8.* All data are presented as means ± SD from at least three independent experiments. Different letters in the lower case represent significant difference among groups (*p* < 0.05).

To identify the novel targets and pathways preliminarily, RNA sequencing of HepG2 cells was performed. Transcriptomic analysis of ILA-treated HepG2 cells identified 131 differentially expressed genes (DEGs), comprising 84 upregulated and 47 downregulated genes (Figure 6A). KEGG pathway enrichment analysis of these DEGs revealed that it was significantly enriched in non-alcoholic fatty liver disease (Figure 6B). Notably, the upregulated genes were the components of CII, III, and IV in mitochondria, which are functionally annotated to oxidative phosphorylation. Concurrently, the expression of pro-inflammatory cytokine IL-8 was downregulated (Figure 6C). To further investigate the regulatory network, a protein–protein interaction (PPI) analysis was performed. The resulting network highlighted CYP1A1 as a central node interacting with UGT1A9 and mediated the upregulation of COX5B, a structural subunit essential for the assembly of complex IV (Figure 6D). Quantitative RT-PCR confirmed the upregulation of these key genes (Figure 6E), collectively supporting the activation of the AHR-COX5B axis. These results indicate that ILA alleviates mitochondrial oxidative stress, at least in part, by enhancing oxidative phosphorylation.

### 3.6. Restoration of Insulin Signaling via the IRS-1/PI3K/AKT/GLUT-4 Pathway

**Figure 7:**
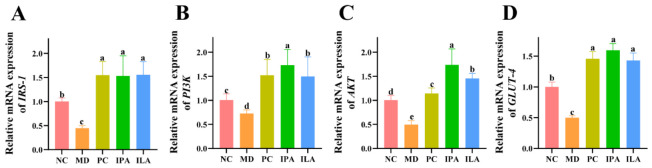
Relative mRNA expression of (**A**) *IRS-1*, (**B**) *PI3K*, (**C**) *AKT*, and (**D**) *GLUT-4*. All data are presented as means ± SD from at least three independent experiments. Different letters in the lower case represent significant difference among groups (*p* < 0.05).

To mechanistically link the alleviation of oxidative stress with enhanced insulin signaling, the expression of key molecules in the insulin pathway was examined. In IR-HepG2 cells, ILA treatment markedly elevated the mRNA levels of *IRS-1*, *PI3K*, *AKT*, and *GLUT-4* (Figure 7A–D). These results suggest that the amelioration of mitochondrial oxidative stress facilitates reactivation of the canonical IRS-1/PI3K/AKT signaling cascade, thereby promoting the expression of the glucose transporter GLUT-4 and restoring cellular insulin sensitivity.

## 4. Discussion

IR is a central pathogenic factor in metabolic syndrome, and accumulating evidence suggests that interventions by gut microbiota are a promising strategy to ameliorate metabolic disturbances [25]. Consistent with the previous studies that supplementation with specific *Lactobacillus* can reverse microbiota dysbiosis and modulate derived metabolites to improve IR [26,27,28], our study demonstrated that oral administration of *L. johnsonii* Y1 improves systemic insulin sensitivity and glucose tolerance, accompanied by a remodeling of gut microbial composition and function. Metabolomic profiling further revealed that tryptophan metabolism was the most prominently altered pathway, aligning with the growing recognition of microbial tryptophan-derived metabolites, such as ILA and IPA, as crucial regulators of host metabolism [29]. Among these, ILA, as a downstream product of tryptophan and an upstream precursor of IPA, has been implicated in metabolic stabilization and IR improvement [10,30].

Recent studies have pointed out that most *Lactobacillus* lack the genes *fldbc* and *acda*, which are responsible for converting ILA to IPA [31]. These findings prompted an investigation into the genome of *L. johnsonii* Y1. Similar to most *Lactobacillus*, *L. johnsonii* Y1 lacks the genes *fldbc* and *acda*, which explains its inability to produce IPA. This observation refines our initial hypothesis that *L. johnsonii* Y1 may confer metabolic benefits primarily through ILA production, despite its inability to generate IPA. Consistent with this hypothesis, the upstream pathway for ILA production appears intact. The biosynthesis of ILA is primarily dependent on the *arat* and *ldh* (sometimes annotated as *fldh*) genes [32], genomic examination has confirmed the presence of these genes in *L. johnsonii Y1*. Notably, we observed multiple gene copies encoding the same protein, which are distributed across distant loci. This phenomenon is not uncommon, as gene duplication serves as a strategy that enables bacteria to enhance gene dosage or maintain functional redundancy, thus providing a selective advantage in fluctuating environmental conditions [33,34]. Additionally, horizontal gene transfer may introduce extra gene copies into varying genomic contexts, further contributing to the observed scattered distribution pattern [35]. Based on the established biosynthetic models and phylogenetic analysis, the gene pair of *ldh2* and *arat1* was identified as the most likely genetic determinant responsible for ILA production in this strain, providing genetic support for our hypothesis.

Microbiota-derived indoles like ILA are known ligands for the aryl hydrocarbon receptor (AHR) in vitro and in vivo [36]. In this study, quantitative RT-PCR confirmed the upregulation of the key genes (Figure 6E), not only demonstrating the strong induction of the classic AHR target CYP1A1 and providing concrete evidence for the activation of the AHR/CYP1A1 axis by ILA [37], but also supporting the activation of the downstream target COX5B. Mitochondrial oxidative phosphorylation serves as a major source and chief regulator of cellular ROS [38]. Inefficient electron transfer across respiratory chain, especially under impaired CIII/IV activity, can promote single-electron reduction of molecular oxygen and superoxide formation [39,40]. In the present study, exposure to ILA markedly reduced cellular ROS levels, and the PPI network analysis further predicted that ILA would not only activate the AHR/CYP1A1 axis but also upregulate COX5B, a subunit critical for the assembly, stability, and catalytic efficiency of mitochondrial CIV [41]. The reduction in ROS subsequently dampened oxidative stress-associated inflammatory signaling, as evidenced by the decreased expression of *IL-8*. As IL-8 can disrupt insulin signaling by inhibiting the IRS/AKT pathway and *GLUT4* expression [42], its attenuation is metabolically beneficial. Consistently, ILA treatment activated the IRS/AKT axis, upregulated GLUT4 mRNA expression, and ultimately restored cellular glucose uptake. This aligns with the established model in which mitigation of oxidative stress helps restore insulin signal transduction [43].

Collectively, our findings delineate a mechanistic pathway through which *L. johnsonii* Y1 ameliorates IR: the bacterium produced ILA via its *arat1* and *ldh2* genes and, subsequently, the derived ILA activated the AHR/CYP1A1 signaling axis, leading to the upregulation of COX5B and potentially enhancing the activity of mitochondrial complex IV. This mitochondrial modulation attenuated oxidative stress and suppressed IL-8-mediated inflammation, thereby alleviating the inhibition on the IRS/AKT pathway. Consequently, IRS/AKT activation restored *GLUT-4* expression and facilitated an increase in cellular glucose uptake.

Nonetheless, this research has limitations that should be addressed in future work. First, the regulatory effects of *L. johnsonii* Y1 may involve metabolites other than ILA, potentially produced in cooperation with the broader gut microbiome. Second, while genetic evidence strongly supports ILA as the key metabolite, direct enzymatic validation is required. Finally, the elucidated mechanism is primarily based on in vitro models; the in vivo effects of ILA and the translational relevance of this axis require further exploration.

## 5. Conclusions

Through multi-omics analysis, we identified tryptophan metabolism as a key functional pathway, and ILA was identified as a candidate of the crucial derived metabolite of *L. johnsonii* Y1, which was found to be associated with its beneficial effects by upregulating COX5B to enhance CIV activity. This enhancement reduced ROS production and attenuated inflammation, thereby restoring insulin sensitivity. Collectively, our work proposes a novel *L. johnsonii*–ILA–mitochondrial axis and provides mechanistic insights into how a gut microbiota-derived metabolite can improve metabolic homeostasis, highlighting its potential as a therapeutic target for IR.

## Data Availability

All raw data have been deposited under accession numbers PRJNA1467283 (RNA-seq), PRJNA1467286 (16S), and PRJCA064553 (metabolomics). Further inquiries can be directed to the corresponding authors.

## Supplementary Materials

The following supporting information can be downloaded at https://www.mdpi.com/article/10.3390/microorganisms14061231/s1, Figure S1: Cell viability of (A) MET, (B) IPA, and (C) ILA determined by CCK-8.; Table S1: Primers for RT-qPCR; Table S2: Genome features of *L. johnsonii* Y1; Table S3: Genes related to tryptophan catalyzation in *L. johnsonii* Y1.
