# Supplementary material for: Improvement of Insulin Resistance by Lactobacillus johnsonii-Derived Indole-3-Lactic Acid
Source: Microorganisms. 2026 May 30;14(6):1231. doi: 10.3390/microorganisms14061231 (PMC13302831; doi:10.3390/microorganisms14061231)
Supplement: Supplementary file 1 [file microorganisms-14-01231-s001.zip › microorganisms-4299873-supplementary.pdf]

Supplementary Table S1 Primers for RT-qPCR

Gene	Forward primer (5'-3')	Reverse primer (5'-3')
β- ACTIN	ACAGAGCCTCGCCTTTGCC	GATATCATCATCCATGGTGAGCTGG
AHR	TTGGTTGTGATGCCAAAGGA	GACTGGACCCAAGTCCATCG
CYP1A1	GCGCTATGACCACAACCACC	TCCCGGATGTGGCCCTTCT
UGT1A9	TATTTCTCCCTCCCCTCCGT	GGCTGTAGAGATCATACTCCG
COX5B	TGCAAAGAAGGGACTGGACC	GACGACGCTGGTATTGTCCT
IL-8	CGCCAACACAGAAATTATTGTA AA	TTTCCTGGGCAAACTATGTATGG
IRS-1	TCAGTTTCCAGAAGCAGCCA	GAGGTCATTTAGGTCTTCATTCTGC
PI3K	AAATGAAGCCTTTGTGGCTGG	CCATAAGGCAACATCCGAAGA
AKT	CTGCACAAACGAGGGGAGT	GCGCCACAGAGAAGTTGTTG
GLUT-4	ACCAGCATCTTCGAGACAGC	TCCACCAACAACACCGAGAC

Supplementary Table S2 Genome features of *L. johnsonii* Y1.

Characteristic	Chromosome	Plasmid
Topology	Circular	Linear
Seq Length (bp)	2114452	57452
GC content (%)	34.84	35.15
Number of genes	4189	101
Number of rRNA	21	0
Number of tRNA	81	0

Supplementary Table S3 Genes related to tryptophan catalyzation in *L. johnsonii* Y1.

Gene categories	Start-End(bp)	Gene length	Products	AA length
arat	815753-816937	1185	Aspartate aminotransferase	394
	1351803-1352759	957	Aspartate aminotransferase	318
ldh	148615-149586	972	L-lactate dehydrogenase 1	323
	797961-798887	927	L-lactate dehydrogenase 2	308
	2015539-2016552	1014	D-lactate dehydrogenase	337

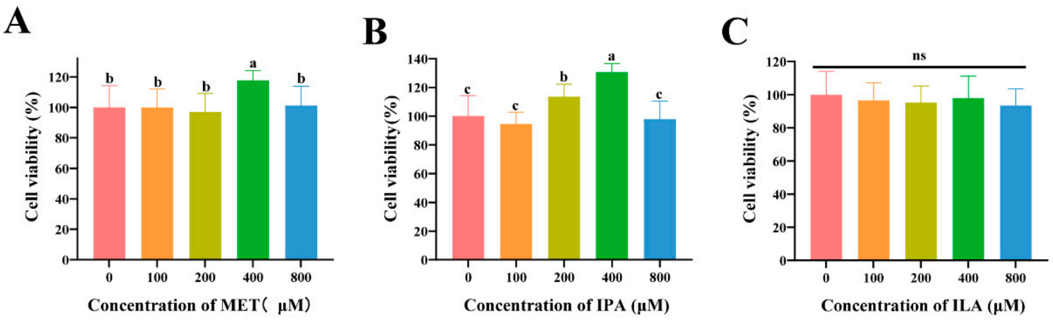

Figure S1 Cell viability of A) MET, B) IPA, and C) ILA were determined by CCK-8. All data are presented as means ± SD from at least three independent experiments. Different letters in the lower case represent significant difference among groups ($p < 0.05$), ns represent none significant difference ($P > 0.05$).
